# Uveitis in spondyloarthritis

**DOI:** 10.1177/1759720X20951733

**Published:** 2020-09-12

**Authors:** Judith Rademacher, Denis Poddubnyy, Uwe Pleyer

**Affiliations:** Department of Gastroenterology, Infectiology and Rheumatology, Charité – Universitätsmedizin Berlin, corporate member of Freie Universität Berlin, Humboldt-Universität zu Berlin and Berlin Institute of Health, Hindenburgdamm 30, Berlin, 10117, Germany; Berlin Institute of Health, Berlin, Germany; Department of Gastroenterology, Infectiology and Rheumatology, Charité – Universitätsmedizin Berlin, corporate member of Freie Universität Berlin, Humboldt-Universität zu Berlin and Berlin Institute of Health, Berlin, Germany; Department of Ophthalmology, Campus Virchow, Charité – Universitätsmedizin Berlin, corporate member of Freie Universität Berlin, Humboldt-Universität zu Berlin and Berlin Institute of Health, Berlin, Germany

**Keywords:** spondyloarthritis, uveitis

## Abstract

Uveitis is the most frequent extra-articular manifestation of axial spondyloarthritis (SpA), occurring in up to one-third of the patients. In the majority of patients, uveitis is acute, anterior and unilateral and presents with photosensitivity, sudden onset of pain and blurred vision. Topical steroids are an effective treatment; however, recurrent or refractory cases may need conventional disease-modifying antirheumatic drugs or biological treatment with monoclonal tumor necrosis factor (TNF) inhibitors, thus also influencing treatment strategy of the underlying SpA.

Though the exact pathogenesis of SpA and uveitis remains unknown, both seem to result from the interaction of a specific, mostly shared genetical background (among other HLA-B27 positivity), external influences such as microbiome, bacterial infection or mechanical stress and activation of the immune system resulting in inflammation. Up to 40% of patients presenting with acute anterior uveitis (AAU) have an undiagnosed SpA. Therefore, an effective referral strategy for AAU patients is needed to shorten the diagnostic delay of SpA and enable an early effective treatment. Further, the risk for ophthalmological manifestations increases with the disease duration in SpA; and patients presenting with ocular symptoms should be referred to an ophthalmologist. Thus, a close collaboration between patient, rheumatologist and ophthalmologist is needed to optimally manage ocular inflammation in SpA.

## Introduction

Axial spondyloarthritis (axSpA), along with psoriatic arthritis (PsA), arthritis associated with inflammatory bowel disease (IBD), reactive arthritis (ReA) and other forms of peripheral SpA, belongs to the heterogenous group of spondyloarthritis (SpA).^1^ The leading symptom is chronic back pain often showing characteristics of inflammatory low back pain with insidious onset, morning stiffness, pain in the second half of the night, and improvement with exercise rather than with rest.^2^ Extra-spinal symptoms include peripheral arthritis, dactylitis and enthesitis. Furthermore, extra-articular (extra-musculoskeletal) manifestations are common, including acute anterior uveitis (AAU), psoriasis and IBD.

The entry criterion for the Assessment in SpondyloArthritis international Society (ASAS) classification criteria for axSpA is a chronic back pain with an onset before the age of 45 years. Furthermore one of the following conditions must be fulfilled: the imaging arm requesting sacroiliitis either on MRI or X-ray and ⩾1 SpA feature (among others, uveitis) or the clinical arm in the case of HLA-B27 positivity together with ⩾2 SpA features.^3^ Patients with a definitive radiographic sacroiliitis fulfilling the modified New York criteria^4^ are classified as radiographic axSpA (r-axSpA; ankylosing spondylitis, AS), while others are classified as non-radiographic axSpA (nr-axSpA). With radiographic progression, nr-axSpA may progress to r-axSpA in about 10% within 2 years.^5,6^

## Epidemiology

AAU is the most frequent extra-articular manifestation in spondyloarthritis; its prevalence ranges between 21% and 33% according to meta analyses.^7–9^ In 2008, a systematic literature analysis reported a prevalence of 32.7% (9757 out of 29,077 SpA patients) with at least one flare of uveitis.^9^

The prevalence of uveitis increases with disease duration. While only 12% of those SpA patients experienced a flare of uveitis within the first 5 years of their disease, 43% of the patients with a long-standing disease (more than 30 years) had an ophthalmological manifestation.^9^ This may explain why patients with r-axSpA have a higher pooled prevalence of uveitis (23%) than patients with non-radiographic SpA (16%), as another meta-analysis of 2236 r-axSpA and 1242 nr-axSpA patients showed.^7^ The prevalence of uveitis also differs between the different subtypes of SpA: it ranges from 37% in arthritis associated with inflammatory bowel disease, 33% in r-axSpA, 26% in ReA, 25% in psoriatic arthritis to 13% in undifferentiated SpA.^9^

Not surprisingly, there is also a higher prevalence of uveitis in HLA-B27 positive SpA patients (40% *versus* 14% in HLA-B27 negative SpA with an odds ratio of 4.2).^9^ Moreover, the prevalence of uveitis varied by region: it was highest in studies from North America (35%) and Europe (29%) compared with Asia (21%) and Latin America (20%).^8^

There are conflicting data regarding the influence of gender: Whereas one meta-analysis reported women to be more often affected by uveitis (prevalence of 33% *versus* 29% in men, odds ratio of 1.3),^9^ other studies reported no difference^10^ or even a higher prevalence in male patients.^11^ Uveitis in SpA is, in the majority of cases, acute (89%), anterior (91%) and unilateral (87%); about 50% of the patients have a recurrent uveitis.^9^ Only a small number (9%) was reported to have bilateral eye involvement (both eyes at the same time), whereas alternating eye involvement occurs frequently in recurrent uveitis flare. Four percent of SpA patients have posterior uveitis.

## Pathophysiology

The exact pathogenesis of SpA, as well as uveitis, remains, to date, unknown. However, there seem to be close links between the two diseases, which are thought to result from the interaction of a specific mostly shared genetic background, external influences such as microbiome, bacterial infection or mechanical stress and activation of the immune system resulting in inflammation.

To date, it is not known why the inflammation takes place in specific sites of the body such as the axial skeleton in axSpA and the eye in uveitis. Several tissue-specific explanations are proposed, but none so far proven:^12^

- Mechanical stress in the entheses or respectively lens and ciliary body;
- Increased HLA-B27 expression;
- Higher susceptibility to either deposition of microbial-associated molecular patterns or ER stress;
- Molecular mimicry between infectious agents and eye or joint patterns;
- Mucosal-like addressins or increased vascular adhesion molecules that both may increase infiltration of immune cells from either the gut or elsewhere.^12^

### Genetics

**Figure 1.**
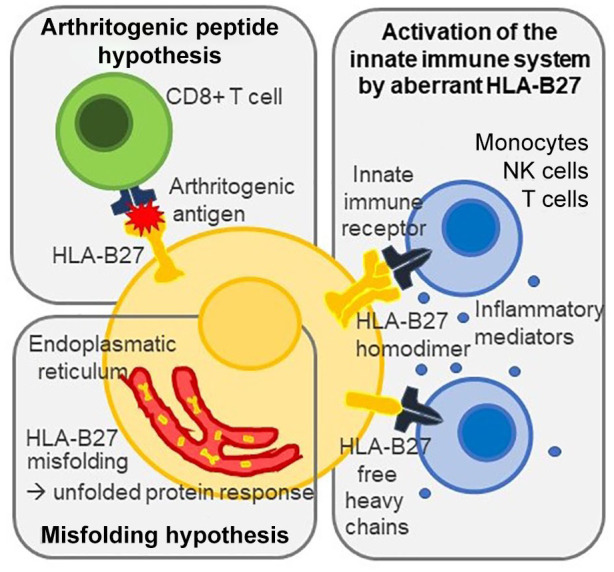
Main hypotheses for the contribution of HLA-B27 to the pathogenesis of uveitis and spondyloarthritis: schematic representation of the arthritogenic/uveitic peptide hypothesis, the HLA-B27 misfolding hypothesis, and the hypothesis of activation of the innate immune system by aberrant HLA-B27. NK, natural killer

There are multiple genes predisposing to SpA as well as to AAU, but HLA-B27 remains the gene with the strongest impact on both diseases. About 50% of AAU and up to 90% of axSpA patients are HLA-B27 positive.^13^ Three main hypotheses provide possible explanations for its contribution to the pathogenesis: the arthritogenic/uveitic peptide hypothesis, the HLA-B27 misfolding hypothesis and the hypothesis of activation of the innate immune system by aberrant HLA-B27 (Figure 1).^1,14^

The physiological function of HLA-B27 is to present processed antigens to cytotoxic CD8+ T cells. According to the first hypothesis, those antigens could become “arthritogenic” or “uveitic” if either they share a sequence homology with self-peptides or they are self-peptides. The respective B27-restrictive CD8+ T cells will thus become autoreactive and able to induce inflammation in the joint or eye, leading to arthritis and uveitis. First evidence was reported back in the 1990s, when autoreactive CD8+ T cell clones were derived from the synovial fluid of ReA and AS patients.^15^ HLA-B27 restricted T cells that recognize both peptides derived from intracellular bacteria such as *Chlamydia, Yersinia* and *Salmonella* and uninfected healthy cells further supported that hypothesis.^15–17^ In addition, multiple studies reported elevated levels of serum antibodies to Gram-negative bacteria such as *Chlamydia, Yerisinia, Proteu*s and *Helicobacter pylori* in patients with AAU.^18^ Contrary to most other HLA-B27 subtypes, HLA-B*27:06 and *27:09 are not associated with AAU and SpA. Recent studies therefore compared peptide binding of the different HLA-B27 subtypes: although no qualitative difference was found,^19^ 26 peptides which presented in higher abundance by disease-related HLA-B27 subtypes were identified as possible arthritogenic peptides.^20^

Another theory is based on the fact that HLA-B27 tends to misfold and thus trigger the so-called unfolded protein response,^21^ an endoplasmatic reticulum (ER) stress, which leads to the production of proinflammatory cytokines. In transgenic rats, HLA-B27 misfolding was shown to be able to induce interleukin (IL)-23 induction and thus activate the IL-23/IL-17 axis, known to be involved in the pathogenesis of SpA.^22^ Moreover, ER stress seems to counteract the immunosuppressive effects of IL-10 by inhibiting STAT3 activation.^23^

The third hypothesis also relies on non-antigen-presenting functions of HLA-B27 and defines SpA and uveitis as diseases with autoinflammatory mechanisms.^24^ HLA-B27 is able to form homodimers, which may then be presented on cell surface following endosomal recycling.^25^ Cell surface B27 homodimers as well as B27 free heavy chains bind to innate immune receptors on natural killer cells, T cells and monocytes, and were shown on peripheral blood mononuclear cells (PBMCs) and synovial cells of patients with SpA.^26^ Thus, HLA-B27 may lead to the activation and production of pro-inflammatory mediators *via* the innate immune system independent of antigens. Moreover, antigen presenting cells (APCs) expressing B27 homodimers were shown to stimulate IL-17 production of CD4+ T cells *via* the killer-cell Ig-like receptor KIR3DL2 in SpA.^27^ A decreased KIR-inhibition was described in B27-associated AAU with and without axSpA, which may explain the rapid onset of intraocular inflammation in AAU.^28^

Besides MHC alleles, other genes such as endoplasmic reticulum aminopeptidase-1 (which trims peptides in the ER before loading the processed peptides onto MHC1 molecules,) and IL-23-R are known to be associated with both SpA and AAU (Table 1).^13,14,29^

**Table 1.** Clinical features and genetic background of HLA-B27 AAU (modified from Wakefield *et al.*^14^).

Association of AAU	Genetic associations	Clinical features
Idiopathic	HLA-B27 ERAP-1 EYS	Most often unilateral > bilateral and AAU or recurrent disease. Up to 50% of patients with AAU are HLA-B27-positive.
Axial spondyloarthritis	HLA-B27 ERAP-1 IL- 23R IL-6R Intergenic region 2p15 Chr. 1q32- KIF21B	AAU or RAAU occur in approximately 35% of patients with AS and increase with increasing age.
Reactive arthritis/peripheral spondyloarthritis	HLA-B27	AAU in 60% of patients. Severe AAU/CAU, especially associated in immune compromised individuals (HIV infection). Rarely posterior uveitis and retinal vasculitis.
Psoriatic arthritis	Cw6 HLA-B27	AAU, RAAU, and BAAU, less common than in AS. 15% of patients have AAU.
Inflammatory bowel disease	IL-10–19 IL-18R-ILR-1 HLA-B27	AAU, RAAU, or BAAU, in <10% of patients with IBD. Occasionally severe disease with posterior uveitis and retinal vasculitis.

AAU, acute anterior uveitis; AS, ankylosing spondylitis; BAAU, bilateral acute anterior uveitis; CAU, chronic anterior uveitis; ERAP, endoplasmic reticulum aminopeptidase-1; IBD, inflammatory bowel disease; RAAU, recurrent AAU

Genes such as IL-10, IL-18R1 and EYS are associated only with AAU and not with SpA.^13^ Interestingly, IL-10 and IL-18R1 are known to also be associated with inflammatory bowel disease.^30^

### Microbiome

The microbiome is supposed to play a role in the evolution of both SpA and uveitis. This hypothesis is supported by animal models, where HLA-B27 transgenic rats do not develop gut and joint inflammation when kept in a germ-free environment.^31^ Human microbiome studies using 16s RNA sequencing described an intestinal dysbiosis in axSpA compared with feces of healthy donors^32^ as well as in biopsies from ileum^33^ and colon.^34^ While biodiversity was reduced in feces, the biopsies showed an increased diversity. Metagenomic analysis also described a distinctive microbiome in AS patients with a higher abundance of *Prevotella* species and a lower *Bacteroides spp.* together with a reduced overall diversity.^35^ A recent study confirmed the lower abundance of *Bacteroides* and *Lachnospiraceae* together with a higher abundance of *Proteobacteria, Enterobacteriaceae, Bacilli, Streptococcus* species and *Actinobacteria* in SpA.^36^ Different bacterial species were reported to be elevated in SpA, among those *Ruminococcus gnavus*^32^ and *Dialister*, which showed a correlation with disease activity.^34^ Moreover, fecal microbiome was different between patients with normal and elevated fecal calprotectin, which indicates gut inflammation.^36^ For AAU, only scarce data exist and do not so far indicate a distinct microbiome phenotype.^37^

But how does intestinal dysbiosis lead to joint and eye inflammation? A common hypothesis is based on the fact that the gut mucosal immunity and barrier function is disturbed in SpA and AAU. Up to 60% of the patients with SpA have subclinical gut inflammation in the ileum and/or caecum.^38^ Recently, adherent and invasive bacteria were found in the inflamed gut of AS patients.^39^ Bacteria were able to increase zonulin expression, which may alter endothelial tight junctions and lead to a damage of the intestinal mucosal and the gut vascular barriers.^39^ Thus, intestinal-derived proteins and bacterial products may translocate *via* the blood to the joint and eye and lead to immune response and inflammation. Bacterial products were described in the joints in ReA,^40^ but not in the anterior chamber.

Other hypotheses of the microbiome’s contribution to the pathogenesis include molecular mimicry between bacteria such as *Klebsiella* and HLA-B27^41^ or the translocation of intestinal immune cells to the eye and joint. Leukocyte trafficking between the gut and the eye as extra-intestinal tissue was shown in the animal model of experimental autoimmune uveitis (EAU).^42^ Furthermore, in EAU, increased gut permeability and antimicrobial peptide expression together with increased effector T cells in the mesenteric lymph nodes preceded the ocular inflammation.^43^

## Ophthalmologic manifestations of SpA

Extra-articular involvement in SpA is common and affects the eye (intraocular inflammation as uveitis), gut (inflammatory bowel disease), skin (psoriasis) and parenchymal organs. Eye involvement remains the most frequent extra-articular manifestation, present in one-third of SpA patients.^8^ The presentation is so typical that uveitis has been included as an important clinical feature and belongs to the classification criteria for axial and peripheral SpA.^3^ Other ocular manifestations, such as posterior uveitis, episcleritis and scleritis, may occur but are much less frequent.

**Figure 2.**
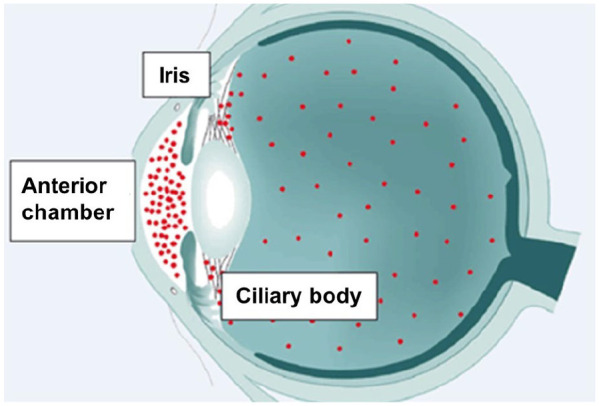
Schematic illustration. Based on the part of the uvea involved, uveitis can be anterior (involving the anterior chamber and iris), intermediate (involving the ciliary body, vitreous) and posterior (involving the choroid) or panuveitis (involving all the parts).

Uveitis is defined by intraocular inflammation, which includes the iris, ciliary body and choroid. It is further classified into anterior, intermediate, posterior and panuveitic forms based on the primarily affected anatomical compartment of the eye.^44,45^ This classification is clinically important, since it assists in the differential diagnosis of its etiology and treatment approach. AAU as inflammation of iris and the ciliary body (Figure 2) is by far the most frequent manifestation, affecting approximately 60–90% of uveitis cases. The fundamental pathophysiological and immunological mechanisms of intraocular inflammation are still poorly understood. Of particular interest may be the intraocular milieu, for example, in the aqueous humor of the eye. The expression of Th1, Th17 and Th2 auto-reactive lymphocytes is considered important in the pathogenesis of intraocular inflammation. So far, only a few studies on the aqueous humor composition of corresponding mediators are available. When comparing HLA-B27-associated AAU and idiopathic AAU with non-inflamed controls, there was a significant increase of inflammatory mediators. Interestingly, however, there was no significant difference in mediator composition with respect to IL-1 (IL-1α, IL-1β, IL-1RA), IL-18 and IL-36RA when comparing HLA-B27 positive individuals with AAU and “idiopathic” AAU.^46^

## Diagnostic

### AAU clinical features

**Figure 3.**
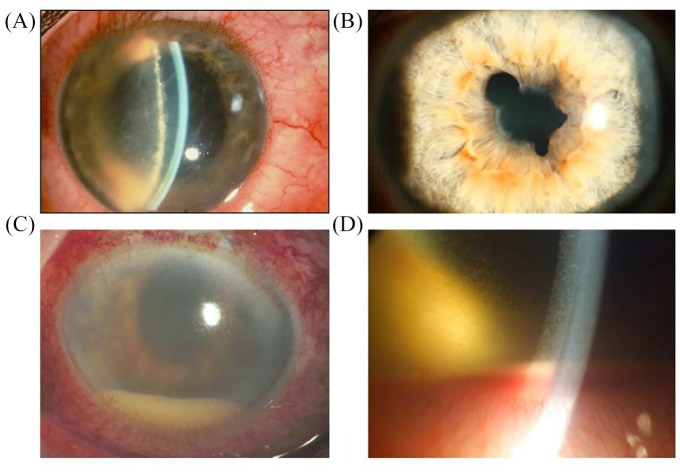
Photographs of slit lamp findings on acute anterior uveitis examination. (A) Ciliary injection (“red eye”) with fibrin exudation at the pupil margin which often leads to (B) “posterior synechiae” (the iris is bound to the lens, leading to irregular pupil margins). (C, D) Severe anterior segment inflammation with deep and superficial dilatation of vessels, “hypopyon” and fibrin coating on the front of the lens. Hypopyon indicates severe breakdown of the blood–iris barrier, leading to intraocular exudation of leukocytes that settle in the anterior chamber of the eye due to gravity. (All clinical figures: Department of Ophthalmology, Campus Virchow, Charité.)

There are a variety of clinical symptoms and findings that are characteristic of ocular involvement in SpA. This is especially true for AAU. Characteristic is the acute onset of intraocular inflammation with focus on the iris and ciliary body. In SpA patients, intraocular involvement occurs typically as unilateral recurrent AAU, even though rarely bilateral inflammation may occur (Figure 3 and Table 2).

**Table 2.** Typical symptoms of uveitis in spondyloarthritis.

– Prodromal stage (“organ sensation”)
– Sudden onset
– Strictly unilateral, but may “flip-flop”
– Pain, redness
– Photosensitivity

#### Symptoms

Patients may report on a possible prodromal stage of a few hours to a day that is best described as “organ sensation” (“I feel my eye”) followed by photosensitivity, and sudden onset of pain in the affected eye (Table 2). Photophobia is caused by ciliary muscle spasms, which are also responsible for pain and are associated with anterior chamber cellular inflammation. Pain, circumlimbal redness and blurred vision are common symptoms. The reduction in vision can be mild to severe.

#### Signs

Conjunctival and ciliary injection lead to visible “redness” of the affected eye. At the same time there is inflammation induced “miosis” (narrow pupil). Both findings are already macroscopically recognizable and visible to the non-ophthalmologist. For further assessment of intraocular inflammation, the slit-lamp examination is indispensable. This allows differentiation and quantification of intraocular inflammation. Based on the Standardization of Uveitis Nomenclature (SUN) classification, the severity of inflammation can be quantified by intraocular cells and presence of protein in the anterior chamber and aqueous humor.^44^ In addition, complete breakdown of the blood–iris barrier may lead to direct leukocyte sedimentation into the anterior chamber (“hypopyon”) (Figure 3). Another frequent finding in SpA-associated AAU is fibrin exudation. This can lead to the formation of “posterior synechiae” as a secondary sequela, resulting in adhesions between the iris and the lens (Figure 3).

As a discriminating feature in SpA-associated AAU, intraocular pressure (IOP) is often reduced in the affected eye compared with the fellow eye. This may assist in the differential diagnosis against, for example, virus-associated intraocular inflammation with often significantly increased IOP. It is assumed that the intraocular release of prostaglandins is responsible for this pressure drop.

**Figure 4.**
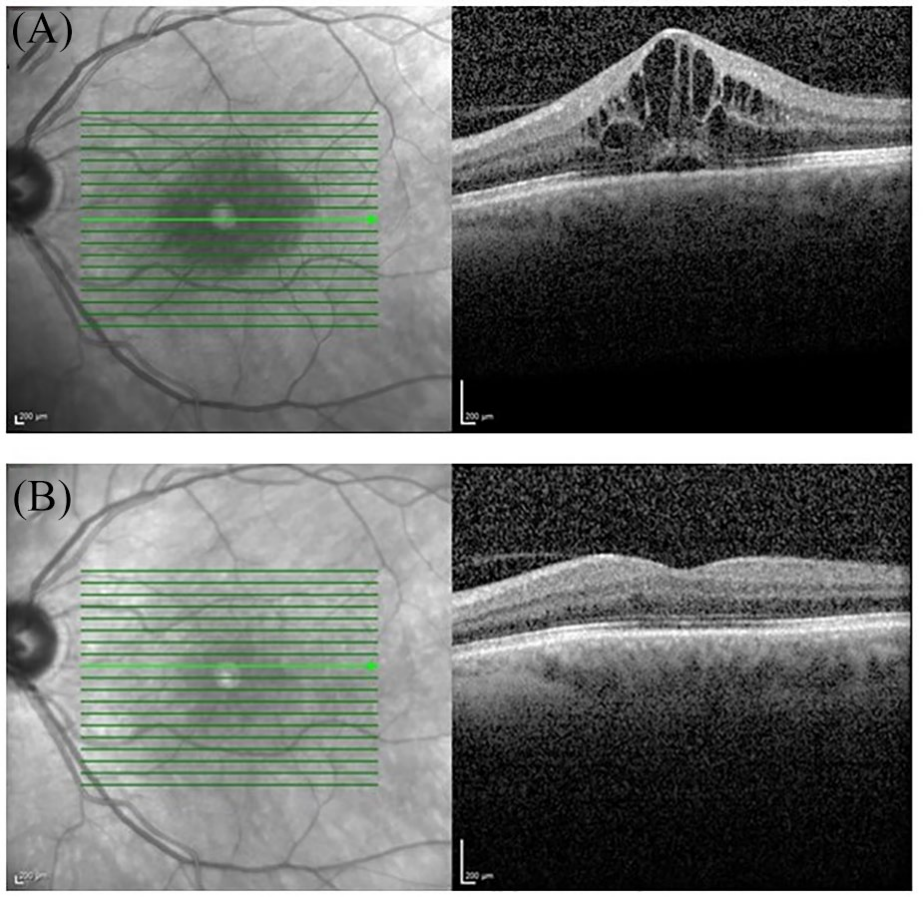
Optical coherence tomography (OCT) in uveitic macula edema. (A) Cystoid macular edema and serous retinal detachment on a cross-sectional macular OCT scan. (B) Resolution of cystoid spaces and subfoveal fluid following treatment. Vision improved from 0.5 (A) to 1.0 (B). OCT is a non-contact non-invasive technique that allows *in vivo* imaging of the retina, choroid, optic nerve head, retinal nerve fiber layer and the anterior structures of the eye.

Although the focus of inflammation is within the anterior compartment of the eye, involvement of the posterior segment may occur, likely through diffusion of immune mediators. This may even occur at the first episode of AAU and result in extracellular fluid accumulation in the macula area presenting as macular edema. It often has an impact on vision and manifests for the patients with so-called metamorphopsia (perceptual distortion of linear objects). Non-invasive diagnostics using optical coherence tomography (OCT) now enable rapid diagnosis and follow-up of this sight threatening complication (Figure 4).

Typically, the course of the AAU ends spontaneously within a few weeks. Rosenbaum observed SpA-associated AAU as predominantly unilateral in 52% of his patients with another 42% as a “flip-flop” manifestation. In contrast, simultaneous inflammation of both eyes remains an exception.^47–49^ Recurrences with a very variable inflammation-free interval (from weeks to years) are typical. AAU is differentiated from chronic acute uveitis, which by definition persists longer than 3 months.

#### Differential diagnosis

Anterior uveitis may be caused by a broad spectrum of etiologies that include both non-infectious as well as infectious disorders (Table 3). Interestingly, SpA is not unique in being related to both uveitis and arthritis. Patients with IBD, such as ulcerative colitis or Crohn’s disease, often have a chronic and persistent course of anterior uveitis. In addition, ReA and postinfectious conditions caused, for example, by spirochetae (Lyme disease, syphilis) or *Tropheryma whipplei* need to be considered. Furthermore, Behcet’s disease and sarcoidosis are differential diagnoses often involving joints, eyes and other organ systems (skin, lung or central nerve system). In these conditions, and in contrast to SpA-associated AAU anterior uveitis more commonly both eyes are affected simultaneously and the condition runs a more chronic clinical course.

**Table 3.** Differential diagnoses of acute anterior uveitis.

Differential diagnosis	Clinical findings	Further diagnostic and procedures
AU associated with SpA	− Unilateral–alternating (“flip-flop”) − Non-granulomatous inflammation (fibrin!) − Often synechiae − Symptoms of SpA: inflammatory back pain	− HLA-B27 − Inflammatory parameters − Rheumatological evaluation − X-ray/MRI of the sacroiliac joints
AU associated with reactive arthritis	− AAU in 60% of ReA patients − Often severe AAU/CAU − Signs/symptoms of urethritis	− HLA-B27 − Inflammatory parameters − Gram-negative bacteria (*Chlamydia, Shigella*) − Rheumatologist, urologist
Morbus Behçet	− May initially present as AAU hyperacute with “cold hypopion” − frequent: occlusive retinal vasculitis (angiography!) − Symptoms/signs of general illness: recurrent oral/genital apthen (> 95%), joint, skin and CNS involvement	− HLA-B51 − Inflammatory parameters − Dermatologist, rheumatologist, neurologist
Vogt–Koyanagi–Harada disease	− AAU/CAU often simultaneously bilaterally − Granulomatous KPs − Mainly: panuveitis (ocular fundus exam!) − Papillitis: “Sunset glow fundus” − Symptoms/signs of general illness: meningism (malaise, fever) headache, back and neck stiffness − Tinnitus − Skin: alopecia, poliosis, vitiligo	− HLA-DR4/DRB1*04 − Pleocytosis in cerebrospinal fluid − Dermatologist, internist, neurologist
Sarcoidosis	− AAU/CAU often simultaneously bilaterally − Granulomatous KPs − Often iris granulomas (“Koeppe nodules”), rarely iris atrophy − Frequent: panuveitis (ocular fundus exam!) − Symptoms/signs of general illness (skin, lung, liver)	− Chest X-ray/CT scan − Lab test: IL-2R, ACE; calcium and phosphate in serum; serum lysozyme − Pulmonologist
TINU syndrome	− Probably underdiagnosed − AAU/CAU often simultaneously bilaterally − Non-granulomatous inflammation Symptoms/signs of general illness: − Nephritis, proteinuria, fever	− Lab test: proteinuria, ß2 microglobulin in urine − Nephrologist
Syphilis	− “Great imitator”! − Uni/bilateral AAU–panuveitis − Symptoms/signs of general illness: primary – chancre; secondary – skin rash; tertiary – malaise, joint, CNS involvement	− Lab test: TPPA, FTA-ABS, VDRL, HIV test − Lumbar puncture − Dermatologist, internist, neurologist
Borreliosis	− Uni/bilateral AAU–panuveitis; (probably overdiagnosed) − History: insect/tick bite − Symptoms/signs of general illness: erythema migrans; joint, CNS involvement	− Lab test: ELISA, immunoblot for IgG and IgM − Dermatologist, internist, neurologist
Tuberculosis	− AAU/CAU mainly bilaterally − Granulomatous KPs; often iris granulomas − Frequent: panuveitis, choroiditis (ocular fundus exam!) − History – exposure? Immune deficient?	− Lab: quantiferon-test, HIV − Chest CT scan − Sputum diagnosis − Pulmonologist
Herpes virus-associated AAU	− Mainly unilateral (non-alternating) − Non-/granulomatous inflammation − Elevated IOP, iris defect, non/granulomatous KPs − VZV: history of dermatomal vesicular rash	− Antibody detection (PCR) for CMV, HSV, VZV in aqueous humor

AU, anterior uveitis; AAU, acute anterior uveitis; ACE, angiotensin converting enzyme; CAU, chronic anterior uveitis; CMV, cytomegalovirus; CNS, central nerve system; CT, computed tomography; ELISA, enzyme-linked immunosorbent assay; FTA-ABS, Fluorescent Treponemal Antibody-Absorption test; HSV, herpes simplex virus; HIV, human immunodeficiency virus; IL, interleukin; KP, corneal endothelial precipitate; MRI, magnetic resonance imaging; NSAID, non-steroidal anti-inflammatory drug; PCR, polymerase chain reaction; ReA, reactive arthritis; SpA, spondyloarthritis; TINU, tubular interstitial nephritis with uveitis; TPPA, treponema pallidum particle agglutination assay; VDRL, venereal disease research laboratory; VZV, varicella zoster virus.

Infectious causes of AAU remain an important differential diagnosis, since therapy fundamentally differs. Virus-associated inflammation, in particular caused by herpes viruses, is among the most common etiologies of anterior uveitis (AU). There are some typical clinical findings, which may already point towards this etiology. Intraocular inflammation usually presents as unilateral AAU but without “flip-flop” occurrence. Frequent findings include increased IOP due to concomitant trabeculitis, iris stromal defects and corneal endothelial precipitates. Intraocular detection of viral genome by PCR or viral antibody detection may support the diagnosis in unclear cases.^50^ In most patients, herpes viruses remain latent in neural ganglia and may result in reactivation of AU. Since an exhaustive discussion of the differential diagnosis of AAU is beyond this article, the most common causes are summarized in Table 3.

### Evaluation of intraocular inflammation

Slit lamp evaluation allows not only classification of anatomical discrimination of uveitis, but also graduation of inflammatory intensity in the anterior chamber. Inflammatory cells and protein exudation can be directly visualized. Interestingly there are attempts to further enhance the differentiation of cell subtypes using OCT techniques, allowing the identification of monocytes, lymphocytes, granulocytes and red blood cells (Figure 4).

In addition, unlike any other medical field, the subtype of inflammation can be assessed directly, for example, distinguishing between granulomatous and non-granulomatous inflammation. This further enhances the differential diagnosis and precision of clinical observation.

Although in the majority of patients AAU runs a benign course, complications may occur. Several studies indicate a higher frequency of secondary findings and complications in HLA-B27+ patients with axSpA such as synechiae and secondary glaucoma. In addition, visual outcome was poorer, probably due to a higher rate of secondary cataracts in HLA-B27+AAU patients with AS compared with HLA-B27+ AAU without AS and more frequent involvement of the posterior eye segment, particularly cystoid macular edema.^51^

### Screening uveitis patients for SpA

Correct and early diagnosis is an important prognostic factor in SpA.^52^ Early diagnosis and early treatment initiation are associated with a better treatment response and may be able to retard the development of structural damage.^53–55^ The delay between symptom onset and diagnosis (5–10 years) remains one of the highest in rheumatology and has not decreased over the past years.^56,57^ Both the patient and general practitioner often classify back pain as a non-specific complaint. The ophthalmological manifestation may therefore be the first occasion for the consultation of a specialist. An effective screening of patients with AAU could therefore enable an early diagnosis of an underlying SpA. This seems to be especially important, as the diagnostic delay was reported to be longer in patients with uveitis (10.9 *versus* 5.9 years).^58^ Furthermore, AAU was shown to affect physical aspects of the quality of life as measured by the SF-36, especially in patients with a so far undiagnosed SpA.^59^

Different approaches have been proposed to decide which AAU patients should be referred to the rheumatologist for further diagnostic work-up: Feltkamp *et al*. recommended assigning all HLA-B27 positive AAU patients to the rheumatologist.^60^ Recently, besides HLA-B27, other factors (higher C-reactive protein, male gender and young age) were reported to be predictive for SpA in AAU patients.^61^ However, ophthalmologists only rarely perform laboratory diagnostics. In addition, AAU can be controlled relatively well with topical steroids and long intervals between uveitis relapses may delay further diagnostic work-up.

The Spanish SENTINEL study analyzed 798 AAU patients with either HLA-B27 positivity or recurrent disease for undiagnosed SpA and found a prevalence of 50% for axSpA and 17.5% for peripheral SpA; in both cases the prevalence was even higher in HLA-B27 positive patients.^62^ Another study performed MRI of spine and sacroiliac joints in 73 AAU patients with chronic back pain with onset prior to the age of 45 and found a prevalence of 20% of axSpA.^63^

Because of its fundamental importance for the early detection of underlying disorders, structured algorithms have been proposed for early recognition of SpA in AAU patients. One of the recent attempts is the “DUET – Dublin Uveitis Evaluation Tool”.^64^ Patients with AAU should be referred to a rheumatologist, if they have either chronic back pain starting prior to the age of 45 years or joint pain requiring a General Practitioner visit and either HLA-B27-positivity or psoriasis. In this cohort 42% of patients with AAU had undiagnosed SpA.^64^ With the DUET test-algorithm a sensitivity of 96% with a specificity of 97% was achieved.

Most recently, the Assessment of SpondyloArthritis international Society (ASAS) proposed a referral algorithm for early identification of patients with a high probability of axSpA by non-rheumatology specialists.^65^ According to this tool, patients with AAU should be referred to a rheumatologist in the presence of chronic back pain starting prior to 45 years of age. This referral instrument is being currently evaluated prospectively.

In any case, a close collaboration between ophthalmologist and rheumatologist is required in order to improve early diagnosis of SpA manifesting with AAU.

### Screening SpA patients for ophthalmological manifestations

Although there is a high prevalence of uveitis in AS, knowledge of the underlying risk factors leading to the development of uveitis in these patients is insufficient. Predictive parameters or laboratory findings for ocular involvement are largely unknown. In addition, clinical symptoms and findings may be non-specific and misleading to the rheumatologist. The broad differential diagnoses associated with a “red eye” indeed can be difficult and may result in misdiagnosis. SpA patients complaining of ocular symptoms should therefore be seen by an ophthalmologist. There may be also a difference amongst types of clinical centers. The incidence of AAU in SpA patients of a university medical clinic has been reported to be lower compared with SpA specialized centers, perhaps due to underdiagnosis.^58^

Factors such as age of onset, gender, duration of disease and HLA-B27 positivity have been suggested to be associated with the clinical course of SpA but not equally proven.^66–68^ Also the time from symptom onset to SpA diagnosis was found to be longer in patients with uveitis^14^ and a higher percentage of patients with uveitis was HLA-B27 positive.^58^

In the search for risk factors and markers for intraocular inflammation in AS different parameters have been discussed. Sun and coworkers were able to correlate increased antistreptolysin levels with the occurrence of intraocular inflammation in AS patients.^69^ In this cohort of 390 patients, hip involvement as well as a higher number of peripheral arthritis was associated with an increased risk for uveitis. This has also been confirmed by previous studies, which reported AS patients with peripheral arthritis^70,71^ and heel pain more likely^72^ to develop AAU.

Furthermore, a strong correlation to the occurrence of AAU was reported for the HLA-B27 genotype. HLA-B27 positivity was associated with a 3.8-fold increased risk for the occurrence of AAU.^73^ However, there are also data that indicated no association with HLA-B27.^14,69^ Besides HLA-B27 further genetic associations have been found to predispose to AAU (see Genetics and Table 1).

A cross-sectional study of 146 Chinese AS patients in Taiwan with a prevalence of uveitis of 16% found a higher Bath Ankylosing Spondylitis Disease Activity Index (BASDAI), Bath Ankylosing Spondylitis Functional Index (BASFI) and decreased physical mobility measured by finger-to-floor, occiput-to-wall distances and Schober test in the patients with concomitant AAU.^74^ However, others found no association between the presence of uveitis and disease activity.^75^

## Treatment options

The management of AAU complicating axSpA should necessarily involve both experts, ophthalmologist and rheumatologist. In addition to condition specific first-line treatments [steroids for uveitis, or non-steroidal anti-rheumatic drugs (NSAIDs) for axSpA] treatment options include medications efficacious for both indications [tumor necrosis factor inhibitors (TNFi), sulfasalazine only for peripheral SpA]. The latter are, however, only used in refractory disease. For each patient, the treatment should therefore be carefully chosen according to the extent of the respective diseases and their manifestations, and ideally be discussed between both experts together with the patient.

Usually, AAU is managed with a treatment crescendo with the aim of a rapid control of ocular inflammation.^76^ In the majority of patients, control is achieved by topical steroids. If uveitis is complicated by posterior eye or bilateral involvement or refractory to topical medication, systemic treatment may be needed. The first step typically involves systemic corticosteroids. A conventional disease-modifying antirheumatic drug (csDMARD) such as methotrexate (MTX) or sulfasalazine can be considered as a second-line treatment, also to prevent recurrent uveitis, as well as biological DMARD (bDMARD, TNFi). However, as the label of TNFi in uveitis treatment is limited to posterior eye segment inflammation, TNFis are off label for AAU. Therefore, the use of TNFi in AAU patients is guided by either an underlying systemic disorder or a case-to-case decision with off label indication by the ophthalmologist.

The management of axSpA relies on the recent 2019 update of the American College of Rheumatology / Spondylitis Association of America / Spondyloarthritis Research and Treatment Network (ACR/SAA/SPARTAN) recommendation^77^ and the European ASAS/European League Against Rheumatism (EULAR) recommendation of 2016.^78^ The ACR recommendations include separate guidelines for AS-related disorders, among others for patients with recurrent uveitis (PICO 29): for these patients treatment with monoclonal antibodies against TNF is recommended over treatment with other biological therapies.^77^ According to the EULAR guidelines, TNFis are “in current practice” used as first bDMARD, as there is more evidence in both quantity and time of follow-up regarding efficacy and safety compared with IL-17 inhibition.^78^ While the guidelines state that the monoclonal antibodies adalimumab, certolizumab and infliximab are efficacious in preventing uveitis flare, no explicit recommendation for patients with uveitis is given.

### Steroids

Since their introduction in the 1950s for the treatment of ocular inflammation, steroids remain the first line therapy for AAU.^79,80^ In the majority of cases, topical administration (prednisolone acetate or dexamethasone eye drops) is able to control the inflammation and cortisone treatment may be tapered and stopped.^81^ In addition, cycloplegic eye drops may be used to prevent or treat synechia.

If uveitis persists despite topical treatment, or severe inflammation involving posterior segments is present, periorbital, sub-conjunctival or intravitreal injections of triamcinolone acetate, fluocinolone or dexamethasone enable the direct application of steroids.^81^ Alternatively, three intravitreal implants are approved for the management of non-infectious posterior uveitis: a dexamethasone insert^82^ and two fluocinolone devices.^83,84^ Especially in bilateral, posterior segment involvement or refractory disease, systemic administration of steroids may be necessary, usually commencing at 1 mg/kg per day orally (intravenous schema also exist) and then tapered over 6–12 weeks.^85^ If prolonged or repeated topical or systemic steroid treatment is necessary, drug-related side effects should be carefully considered, especially eye-specific effects such as ocular hypertension, cataract and glaucoma.^86^ In this case, cortisone-sparing treatment with DMARDs should be started.

With respect to non-ocular manifestations of SpA, steroids may improve peripheral articular involvement such as arthritis or enthesitis. Even though long-term treatment with steroids is not recommended for axial symptoms of SpA, high dose oral prednisolone (50 mg daily over 2 weeks) has been shown to be effective in AS refractory to NSAIDs.^78,87^ However, whether this effect is maintained when prednisolone is tapered still needs to be analyzed and this determination would be important to further evaluate the role of prednisolone in the treatment. Axial inflammation in PsA showed a better response to intra-muscular depot corticosteroid compared with both AS and non-inflammatory back pain, with a greater Ankylosing Spondylitis Disease Acitiviy Score (ASDAS) and BASDAI improvement after 2 weeks, which was maintained until week 4.^88^

### NSAIDs

NSAIDs are the first-line medication in patients with axSpA.^77,78^ Traditional and COX-2 selective NSAIDs are both similarly effective in reducing inflammation, disease activity and pain, and improving function.^89^

However, only scarce data exist about NSAIDs as treatment for AAU. While they are not used for the treatment of acute flares, a recent retrospective case series of 59 patients with recurrent AAU described a reduction of uveitic flares under systemic NSAID treatment (2.8 average flares per year before treatment *versus* 0.5 under NSAID).^90^ However, several limitations of the study have to be taken into account: only four patients had concomitant AS, only one-third were HLA-B27 positive and patients served as their own control, which includes a potential bias such as the phenomenon of regression to the mean.^91^

NSAIDs suppress the production of prostaglandins and thereby decrease the vascular permeability as well as the permeability of the blood–eye-barrier. One hypothesis is that NSAIDs thus prevent antigens from arriving in the anterior chamber, or the deposition of immune complexes, and thereby inhibit the immune response and upcoming flares.^91^ This could also explain why NSAIDs are not as efficacious once the ocular inflammation has already started.

Topical NSAIDs (such as ketorolac 0.5%; nepafenac 0.1%; bromfenac 0.07%) are used perioperative in cataract surgery to suppress ophthalmic inflammation and prevent cystoid macula edema (CME) and mydriasis, though the underlying evidence is limited.^92^ A small case series reports the efficacy of the topical NSAID nepafenac in six patients with uveitic CME; however, three of the six patients received concomitant topical steroids.^93^ A retrospective case series of 281 uveitic CME patients taking topical nepafenac 0.1% reported a modest improvement in both visual acuity and central macular thickness.^94^ Randomized controlled trials are needed to determine the role of topical NSAIDs in the treatment of uveitis CME.

In summary, the role of NSAIDs, both systemic and topical, in the treatment of AU needs to be further clarified.

### csDMARD

csDMARDs (with the exception of sulfasalazine for peripheral arthritis) are not effective in SpA.^95,96^ In AAU, csDMARD may be used, though off-label, in recurrent, refractory or cortisone-dependent cases to control inflammation and spare cortisone. Sulfasalazine was shown to reduce recurrences and severity of AAU in AS patients in a small randomized controlled trial (RCT) of 22 patients^97^ and to reduce AAU flares in the first year of treatment compared with the year before treatment onset.^98^ A prospective study was able to show similar results for MTX in nine AAU patients: uveitic flares were reduced from 3.4 in the year before therapy onset to 0.9.^99^ Another study showed a cortisone sparing effect in addition to a reduced recurrence rate under MTX treatment; cortisone treatment was able to be stopped in all 19 included AAU patients.^100^ In a retrospective analysis, both MTX and sulfasalazine reduced relapse rates in HLA-B27 positive AAU; moreover, methotrexate showed efficacy on macula edema.^101^

In the Systemic Immunosuppressive Therapy for Eye Diseases (SITE) cohort study, the largest retrospective study in non-infectious uveitis to date, patients with AAU were also included.^102^ Immunomodulatory treatment with azathioprine, cyclosporine A, MTX and myclophenolat mofetil (MMF) were compared and steroid sparing was most frequently achieved under MMF and MTX.^103^ However, adverse events have to be taken into account and with the availability of effective biologics, today csDMARDs, apart from MTX and sulfasalazine, are rarely used in AAU.

### Biological DMARD

Two different biological treatment options are available for axSpA: TNFα- and IL-17A inhibitors. bDMARD should be prescribed if the disease is active despite the treatment with two different NSAIDs over at least 4 weeks.^78^

TNFis were the first bDMARD approved in axSpA.^104^ Five different TNFis are available today: the fusion protein etanercept,^105^ the monoclonal antibodies adalimumab,^106^ golimumab^107^ and infliximab,^108^ and the fragment of a monoclonal antibody certolizumab pegol.^109^ All TNFis showed comparable response rates with an ASAS20 response (improvement of 20%) of approximately 60% after 12–24 weeks and an ASAS40 response of 40–50%.^110^

Monoclonal TNFis are effective in the treatment of AAU complicating SpA, presumably in both, treating acute flares and preventing recurrence. Evidence is mainly based on retrospective analyses of SpA patients under TNFi, indirect comparison between time before and under treatment of the same patients and observational studies. Despite those limitations, all monoclonal TNFis were shown to reduce AAU flares.

Adalimumab is the only TNFi approved for the treatment of non-infectious uveitis – though only for non-anterior uveitis in adults after the favorable outcome of the phase III trials VISUAL-1^111^ and VISUAL-2^112^ in intermediate, posterior and panuveitis. After the SYCAMORE study showed its efficacy in combination with MTX in juvenile-idiopathic arthritis (JIA), adalimumab was furthermore approved by the European Medicine Agency (EMA) for non-infectious AU in children aged 2–18 years.^113^ Adalimumab showed such a clear superiority over placebo (27% treatment failure under adalimumab *versus* 60% under placebo)^113^ that the trial had to be stopped prematurely. The efficacy in JIA-associated uveitis was confirmed by the randomized French ADJUVITE trial.^114^ The 2019 ACR guideline for JIA-associated uveitis recommends thus the use of monoclonal TNFi, either adalimumab or infliximab.^115^ Adalimumab was shown to reduce uveitis flares by 51% also in adult AU associated with SpA (1250 patients under open-label adalimumab).^116^ A smaller study even found a reduction of 80%.^117^

Also SpA patients under certolizumab pegol showed a reduced uveitis flare rate (three per 100 patient-years) compared with placebo (10 per 100 patient-years) during the 6 months double-blind phase of the RAPID-axSpA trial.^118^ Small case series also described a significant reduction of uveitis flare and preserved visual function in AU under certolizumab.^119,120^ Initial results of the still-ongoing, open-label phase IV C-VIEW trial were recently presented, which included 115 SpA patients with recurrent AAU.^121^ Patients experienced a significant reduction of AAU flare rate under certolizumab in the interim analysis after 48 weeks (64% before *versus* 12% under certolizumab treatment), and AAU flares were shorter under TNFi.^121^

Infliximab is the only TNFi which is intravenously administered. Uveitis flares were reduced in AS patients under infliximab compared with placebo (13 AAU flares per 100 patient-years *versus* 37).^108,122^ Golimumab was shown to be effective in a small multicenter study of 15 SpA patients with uveitis refractory to at least one csDMARD.^123^ Golimumab reduced uveitis flares in the open-label, history-controlled GO-EASY study of 93 AS patients with a positive history of uveitis (11 events per 100 patient-years compared with 2.2 under golimumab).^124^

Etanercept is a recombinant fusion protein which, unlike other TNFis, does not show clear efficacy in uveitis and has even been reported to increase the risk of uveitis flares. Indirect comparisons between etanercept and other monoclonal TNFis showed higher AU flare rates under etanercept, which is called paradoxical effect. Out of the 1365 AS patients in the Swedish Rheumatology Quality Register, patients under etanercept showed increased AU rates *versus* pretreatment (AU ophthalmologic visits 60 *versus* 42), in contrast to adalimumab and infliximab, which reduced AU rates (14 *versus* 37 for adalimumab, 28 *versus* 46 for infliximab).^125^ Other observational studies have described similar results.^126–128^ Therefore, etanercept is not recommended in SpA patients with uveitis.

The monoclonal IL-17A antibody secukinumab is the first non-TNFi bDMARD licensed in axSpA.^129^ Secukinumab showed by indirect comparison similar ASAS response rates to TNFis (ASAS20 at 16 weeks of 60%),^130,131^ and was also effective in patients with inadequate response to TNFi, though with lower response rates.^131^

A small study in non-anterior non-infectious uveitis described efficacy of intravenous secukinumab in 13 out of 16 patients,^132^ but three clinical trials with subcutaneous secukinumab did not meet their primary end points and showed no difference compared with placebo.^133^ Pooled analyses of the three RCTs of secukinumab in axSpA show total uveitis flares of 10 out of 721 patients under secukinumab (1.4%) *versus* two out of 269 patients in the placebo group (0.7%; *p* = 0.53).^77,129,134^

The ACR treatment recommendation for axSpA patients with recurrent uveitis advises treatment with TNFi monoclonal antibodies over treatment with other biologics (PICO 29).^77^ This recommendation is based on indirect comparison of the AAU flares in seven observational studies with adalimumab, infliximab, etanercept,^125–127,135–138^ one study with pooled data from four RCTs and three observational studies of infliximab and etanercept^122^ and the RCTs of secukinumab.^129,134^ The latter showed no difference between secukinumab and placebo. Patients under treatment with either adalimumab or infliximab showed the lowest rates of uveitis flares. Treatment with etanercept did not change the flare rate or even increased AAU flares. A recent retrospective study including all four available monoclonal TNFis showed a remarkable reduction of AAU flares during a long-term follow-up period.^120^

### Emerging drugs

Further drugs targeting the IL-17 pathway are in advanced development for the indication of axSpA.^95^ Janus kinase (JAK) inhibitors, small molecules targeting the intracellular signaling of type I and type II cytokine receptors, show proven efficacy in axSpA. Only limited data exist regarding the efficacy of those emerging drugs for uveitis complicating axSpA. While IL-17 inhibition seems to be not effective in AAU,^133^ a case report of a patient with anterior and intermediate uveitis described a favorable outcome under tofacitinib, a pan-JAK-inhibitor.^139^ A phase II trial of tofacitinib in non-infectious uveitis is currently underway [ClinicalTrials.gov identifier: NCT03580343]. Another phase II trial is investigating the effect of filgotinib, a selective JAK-1 inhibitor, in uveitis [ClinicalTrials.gov identifier: NCT03207815].

Other rather experimental approaches relying on the observed dysbiosis and microbiome alteration involve the administration of probiotics, which was reported effective in a case report of AAU.^140^ The possibility of fecal transplantation in AAU is also under discussion.^141^

## Conclusion

AAU is the most frequent extra-articular manifestation of SpA and may affect disease activity, quality of life and treatment decisions. The clinical presentation of AAU is often typical with unilateral eye involvement, sudden onset, photosensitivity and blurred vision. In the majority of cases, topical steroids are effective; only recurrent or refractory cases might need csDMARD or biological treatment with preferably monoclonal TNF inhibitors.

As up to 40% of AAU patients were shown to have an undiagnosed SpA, AAU provides the possibility for an early recognition of underlying disease. Thus, an effective referral strategy to a rheumatologist is needed to enable early diagnosis and effective treatment.

The core of the management of AAU and SpA is a close cooperation between ophthalmologists and rheumatologists regarding diagnosis, early referral, treatment decision and follow-up.

## References

[1] Sieper J Poddubnyy D Axial spondyloarthritis. Lancet 2017; 390: 73–84. 28110981 10.1016/S0140-6736(16)31591-4

[2] Rudwaleit M Metter A Listing J, et al Inflammatory back pain in ankylosing spondylitis: a reassessment of the clinical history for application as classification and diagnostic criteria. Arthritis Rheum 2006; 54: 569–578. 16447233 10.1002/art.21619

[3] Rudwaleit M van der Heijde D Landewe R, et al The development of assessment of spondyloarthritis international society classification criteria for axial spondyloarthritis (part II): validation and final selection. Ann Rheum Dis 2009; 68: 777–783. 19297344 10.1136/ard.2009.108233

[4] van der Linden S Valkenburg HA Cats A Evaluation of diagnostic criteria for ankylosing spondylitis. A proposal for modification of the New York criteria. Arthritis Rheum 1984; 27: 361–368. 6231933 10.1002/art.1780270401

[5] Sampaio-Barros PD Bertolo MB Kraemer MH, et al Undifferentiated spondyloarthropathies: a 2-year follow-up study. Clin Rheumatol 2001; 20: 201–206. 11434474 10.1007/s100670170066

[6] Poddubnyy D Rudwaleit M Haibel H, et al Rates and predictors of radiographic sacroiliitis progression over 2 years in patients with axial spondyloarthritis. Ann Rheum Dis 2011; 70: 1369–1374. 21622969 10.1136/ard.2010.145995

[7] de Winter JJ van Mens LJ van der Heijde D, et al Prevalence of peripheral and extra-articular disease in ankylosing spondylitis versus non-radiographic axial spondyloarthritis: a meta-analysis. Arthritis Res Ther 2016; 18: 196. 27586785 10.1186/s13075-016-1093-z PMC5009714

[8] Stolwijk C van Tubergen A Castillo-Ortiz JD, et al Prevalence of extra-articular manifestations in patients with ankylosing spondylitis: a systematic review and meta-analysis. Ann Rheum Dis 2015; 74: 65–73. 23999006 10.1136/annrheumdis-2013-203582

[9] Zeboulon N Dougados M Gossec L Prevalence and characteristics of uveitis in the spondyloarthropathies: a systematic literature review. Ann Rheum Dis 2008; 67: 955–959. 17962239 10.1136/ard.2007.075754

[10] Webers C Essers I Ramiro S, et al Gender-attributable differences in outcome of ankylosing spondylitis: long-term results from the outcome in ankylosing spondylitis international study. Rheumatology 2016; 55: 419–428. 26385369 10.1093/rheumatology/kev340

[11] Mitulescu TC Popescu C Naie A, et al Acute anterior uveitis and other extra-articular manifestations of spondyloarthritis. J Med Life 2015; 8: 319–325. PMC4556912 26351533

[12] Rosenbaum JT Asquith M The microbiome and HLA-B27-associated acute anterior uveitis. Nat Rev Rheumatol 2018; 14: 704–713. 30301938 10.1038/s41584-018-0097-2 PMC6597169

[13] Robinson PC Claushuis TA Cortes A, et al Genetic dissection of acute anterior uveitis reveals similarities and differences in associations observed with ankylosing spondylitis. Arthritis Rheumatol 2015; 67: 140–151. 25200001 10.1002/art.38873 PMC4302162

[14] Wakefield D Yates W Amjadi S, et al HLA-B27 anterior uveitis: immunology and immunopathology. Ocul Immunol Inflamm 2016; 24: 450–459. 27245590 10.3109/09273948.2016.1158283

[15] Hermann E Yu DT Meyer zum Buschenfelde KH, et al HLA-B27-restricted CD8 T cells derived from synovial fluids of patients with reactive arthritis and ankylosing spondylitis. Lancet 1993; 342: 646–650. 8103147 10.1016/0140-6736(93)91760-j

[16] Ugrinovic S Mertz A Wu P, et al A single nonamer from the Yersinia 60-kDa heat shock protein is the target of HLA-B27-restricted CTL response in Yersinia-induced reactive arthritis. J Immunol 1997; 159: 5715–5723. 9548516

[17] Kuon W Holzhutter HG Appel H, et al Identification of HLA-B27-restricted peptides from the chlamydia trachomatis proteome with possible relevance to HLA-B27-associated diseases. J Immunol 2001; 167: 4738–4746. 11591805 10.4049/jimmunol.167.8.4738

[18] Otasevic L Zlatanovic G Stanojevic-Paovic A, et al Helicobacter pylori: an underestimated factor in acute anterior uveitis and spondyloarthropathies? Ophthalmologica 2007; 221: 6–13. 17183194 10.1159/000096515

[19] Schittenhelm RB Sian TC Wilmann PG, et al Revisiting the arthritogenic peptide theory: quantitative not qualitative changes in the peptide repertoire of HLA-B27 allotypes. Arthritis Rheumatol 2015; 67: 702–713. 25418920 10.1002/art.38963

[20] Schittenhelm RB Sivaneswaran S Lim Kam Sian TCC, et al Human leukocyte antigen (HLA) B27 allotype-specific binding and candidate arthritogenic peptides revealed through heuristic clustering of data-independent acquisition mass spectrometry (DIA-MS) data. Mol Cell Proteomics 2016; 15: 1867–1876. 26929215 10.1074/mcp.M115.056358 PMC5083097

[21] Mear JP Schreiber KL Munz C, et al Misfolding of HLA-B27 as a result of its B pocket suggests a novel mechanism for its role in susceptibility to spondyloarthropathies. J Immunol 1999; 163: 6665–6670. 10586062

[22] DeLay ML Turner MJ Klenk EI, et al HLA-B27 misfolding and the unfolded protein response augment interleukin-23 production and are associated with Th17 activation in transgenic rats. Arthritis Rheum 2009; 60: 2633–2643. 19714651 10.1002/art.24763 PMC2893331

[23] Hansen IS Schoonejans JM Sritharan L, et al ER stress abrogates the immunosuppressive effect of IL-10 on human macrophages through inhibition of STAT3 activation. Inflamm Res 2019; 68: 775–785. 31227842 10.1007/s00011-019-01261-9 PMC6667425

[24] Ambarus C Yeremenko N Tak PP, et al Pathogenesis of spondyloarthritis: autoimmune or autoinflammatory? Curr Opin Rheumatol 2012; 24: 351–358. 22488076 10.1097/BOR.0b013e3283534df4

[25] Bird LA Peh CA Kollnberger S, et al Lymphoblastoid cells express HLA-B27 homodimers both intracellularly and at the cell surface following endosomal recycling. Eur J Immunol 2003; 33: 748–759. 12616495 10.1002/eji.200323678

[26] Kollnberger S Bird L Sun MY, et al Cell-surface expression and immune receptor recognition of HLA-B27 homodimers. Arthritis Rheum 2002; 46: 2972–2982. 12428240 10.1002/art.10605

[27] Bowness P Ridley A Shaw J, et al Th17 cells expressing KIR3DL2+ and responsive to HLA-B27 homodimers are increased in ankylosing spondylitis. J Immunol 2011; 186: 2672–2680. 21248258 10.4049/jimmunol.1002653 PMC3210561

[28] Levinson RD Martin TM Luo L, et al Killer cell immunoglobulin-like receptors in HLA-B27-associated acute anterior uveitis, with and without axial spondyloarthropathy. Invest Ophthalmol Vis Sci 2010; 51: 1505–1510. 19850842 10.1167/iovs.09-4232 PMC2868435

[29] International Genetics of Ankylosing Spondylitis Consortium (IGAS), Cortes A Hadler J, et al Identification of multiple risk variants for ankylosing spondylitis through high-density genotyping of immune-related loci. Nat Genet 2013; 45: 730–738. 23749187 10.1038/ng.2667 PMC3757343

[30] Jostins L Ripke S Weersma RK, et al Host-microbe interactions have shaped the genetic architecture of inflammatory bowel disease. Nature 2012; 491: 119–124. 23128233 10.1038/nature11582 PMC3491803

[31] Taurog JD Richardson JA Croft JT, et al The germfree state prevents development of gut and joint inflammatory disease in HLA-B27 transgenic rats. J Exp Med 1994; 180: 2359–2364. 7964509 10.1084/jem.180.6.2359 PMC2191772

[32] Breban M Tap J Leboime A, et al Faecal microbiota study reveals specific dysbiosis in spondyloarthritis. Ann Rheum Dis 2017; 76: 1614–1622. 28606969 10.1136/annrheumdis-2016-211064

[33] Costello ME Ciccia F Willner D, et al Brief report: intestinal dysbiosis in ankylosing spondylitis. Arthritis Rheumatol 2015; 67: 686–691. 25417597 10.1002/art.38967

[34] Tito RY Cypers H Joossens M, et al Brief report: dialister as a microbial marker of disease activity in spondyloarthritis. Arthritis Rheumatol 2017; 69: 114–121. 27390077 10.1002/art.39802

[35] Wen C Zheng Z Shao T, et al Quantitative metagenomics reveals unique gut microbiome biomarkers in ankylosing spondylitis. Genome Biol 2017; 18: 142. 28750650 10.1186/s13059-017-1271-6 PMC5530561

[36] Klingberg E Magnusson MK Strid H, et al A distinct gut microbiota composition in patients with ankylosing spondylitis is associated with increased levels of fecal calprotectin. Arthritis Res Ther 2019; 21: 248. 31771630 10.1186/s13075-019-2018-4 PMC6880506

[37] Huang X Ye Z Cao Q, et al Gut microbiota composition and fecal metabolic phenotype in patients with acute anterior uveitis. Invest Ophthalmol Vis Sci 2018; 59: 1523–1531. 29625474 10.1167/iovs.17-22677

[38] Mielants H Veys EM Cuvelier C, et al Ileocolonoscopic findings in seronegative spondylarthropathies. Br J Rheumatol 1988; 27(Suppl. 2): 95–105. 3042080 10.1093/rheumatology/xxvii.suppl_2.95

[39] Ciccia F Guggino G Rizzo A, et al Dysbiosis and zonulin upregulation alter gut epithelial and vascular barriers in patients with ankylosing spondylitis. Ann Rheum Dis 2017; 76: 1123–1132. 28069576 10.1136/annrheumdis-2016-210000 PMC6599509

[40] Siala M Jaulhac B Gdoura R, et al Analysis of bacterial DNA in synovial tissue of Tunisian patients with reactive and undifferentiated arthritis by broad-range PCR, cloning and sequencing. Arthritis Res Ther 2008; 10: R40. 10.1186/ar2398 PMC2453759 18412942

[41] Welsh J Avakian H Cowling P, et al Ankylosing spondylitis, HLA-B27 and Klebsiella. I. Cross-reactivity studies with rabbit antisera. Br J Exp Pathol 1980; 61: 85–91. 6769456 PMC2041543

[42] Nakamura YK Janowitz C Metea C, et al Short chain fatty acids ameliorate immune-mediated uveitis partially by altering migration of lymphocytes from the intestine. Sci Rep 2017; 7: 11745. 10.1038/s41598-017-12163-3 PMC5603543 28924192

[43] Janowitz C Nakamura YK Metea C, et al Disruption of intestinal homeostasis and intestinal microbiota during experimental autoimmune uveitis. Invest Ophthalmol Vis Sci 2019; 60: 420–429. 30695094 10.1167/iovs.18-24813 PMC6353239

[44] Jabs DA Nussenblatt RB Rosenbaum JT; Standardization of Uveitis Nomenclature Working Group. Standardization of uveitis nomenclature for reporting clinical data. Results of the First International Workshop. Am J Ophthalmol 2005; 140: 509–516. 16196117 10.1016/j.ajo.2005.03.057 PMC8935739

[45] Pleyer U Pohlmann D [Anatomy and immunology of the eye]. Z Rheumatol 2017; 76: 656–663. 28710528 10.1007/s00393-017-0344-y

[46] Zhao B Chen W Jiang R, et al Expression profile of IL-1 family cytokines in aqueous humor and sera of patients with HLA-B27 associated anterior uveitis and idiopathic anterior uveitis. Exp Eye Res 2015; 138: 80–86. 26116905 10.1016/j.exer.2015.06.018

[47] Rosenbaum JT Uveitis in spondyloarthritis including psoriatic arthritis, ankylosing spondylitis, and inflammatory bowel disease. Clin Rheumatol 2015; 34: 999–1002. 25953065 10.1007/s10067-015-2960-8

[48] Rosenbaum JT Characterization of uveitis associated with spondyloarthritis. J Rheumatol 1989; 16: 792–796. 2778762

[49] Feltkamp TEW HLA-B27, acute anterior uveitis, and ankylosing spondylitis. Advances in Inflammation Research: The Spondyloarthropathies 1985; 9: 211–216.

[50] Pleyer U Chee SP Current aspects on the management of viral uveitis in immunocompetent individuals. Clin Ophthalmol 2015; 9: 1017–1028. 26089633 10.2147/OPTH.S60394 PMC4467646

[51] Yang P Wan W Du L, et al Clinical features of HLA-B27-positive acute anterior uveitis with or without ankylosing spondylitis in a Chinese cohort. Br J Ophthalmol 2018; 102: 215–219. 28607176 10.1136/bjophthalmol-2016-309499

[52] Seo MR Baek HL Yoon HH, et al Delayed diagnosis is linked to worse outcomes and unfavourable treatment responses in patients with axial spondyloarthritis. Clin Rheumatol 2015; 34: 1397–1405. 25185731 10.1007/s10067-014-2768-y

[53] Sieper J Lenaerts J Wollenhaupt J, et al Efficacy and safety of infliximab plus naproxen versus naproxen alone in patients with early, active axial spondyloarthritis: results from the double-blind, placebo-controlled INFAST study, part 1. Ann Rheum Dis 2014; 73: 101–107. 23696633 10.1136/annrheumdis-2012-203201 PMC3888606

[54] Haroon N Inman RD Learch TJ, et al The impact of tumor necrosis factor alpha inhibitors on radiographic progression in ankylosing spondylitis. Arthritis Rheum 2013; 65: 2645–2654. 23818109 10.1002/art.38070 PMC3974160

[55] Rudwaleit M Listing J Brandt J, et al Prediction of a major clinical response (BASDAI 50) to tumour necrosis factor alpha blockers in ankylosing spondylitis. Ann Rheum Dis 2004; 63: 665–670. 15037444 10.1136/ard.2003.016386 PMC1755042

[56] Feldtkeller E Bruckel J Khan MA Scientific contributions of ankylosing spondylitis patient advocacy groups. Curr Opin Rheumatol 2000; 12: 239–247. 10910174 10.1097/00002281-200007000-00002

[57] Redeker I Callhoff J Hoffmann F, et al Determinants of diagnostic delay in axial spondyloarthritis: an analysis based on linked claims and patient-reported survey data. Rheumatology 2019; 58: 1634–1638. 30903141 10.1093/rheumatology/kez090

[58] Gevorgyan O Riad M Sarran RD, et al Anterior uveitis in patients with spondyloarthropathies in a single US academic center: a retrospective study. Rheumatol Int 2019; 39: 1607–1614. 31342080 10.1007/s00296-019-04386-6

[59] O’Rourke M Haroon M Alfarasy S, et al The effect of anterior uveitis and previously undiagnosed spondyloarthritis: results from the DUET cohort. J Rheumatol 2017; 44: 1347–1354. 28668811 10.3899/jrheum.170115

[60] Feltkamp TE Ringrose JH Acute anterior uveitis and spondyloarthropathies. Curr Opin Rheumatol 1998; 10: 314–318. 9725092 10.1097/00002281-199807000-00006

[61] Mitulescu CT Popescu CC Oprea CL, et al A referable clinical pattern of spondyloarthritis-associated uveitis. Rom J Ophthalmol 2018; 62: 155–161. 30206560 PMC6117525

[62] Juanola X Loza Santamaria E Cordero-Coma M; SENTINEL Working Group. Description and prevalence of spondyloarthritis in patients with anterior uveitis: the SENTINEL interdisciplinary collaborative project. Ophthalmology 2016; 123: 1632–1636. 27084561 10.1016/j.ophtha.2016.03.010

[63] Sykes MP Hamilton L Jones C, et al Prevalence of axial spondyloarthritis in patients with acute anterior uveitis: a cross-sectional study utilising MRI. RMD Open 2018; 4: e000553. 10.1136/rmdopen-2017-000553 PMC5845401 29531779

[64] Haroon M O’Rourke M Ramasamy P, et al A novel evidence-based detection of undiagnosed spondyloarthritis in patients presenting with acute anterior uveitis: the DUET (Dublin Uveitis Evaluation Tool). Ann Rheum Dis 2015; 74: 1990–1995. 24928841 10.1136/annrheumdis-2014-205358

[65] Poddubnyy D van Tubergen A Landewe R, et al; Assessment of SpondyloArthritis international Society (ASAS). Development of an ASAS-endorsed recommendation for the early referral of patients with a suspicion of axial spondyloarthritis. Ann Rheum Dis 2015; 74: 1483–1487. 25990288 10.1136/annrheumdis-2014-207151

[66] Dougados M d’Agostino MA Benessiano J, et al The DESIR cohort: a 10-year follow-up of early inflammatory back pain in France: study design and baseline characteristics of the 708 recruited patients. Joint Bone Spine 2011; 78: 598–603. 21458351 10.1016/j.jbspin.2011.01.013

[67] Sieper J van der Heijde D Landewe R, et al New criteria for inflammatory back pain in patients with chronic back pain: a real patient exercise by experts from the Assessment of SpondyloArthritis international Society (ASAS). Ann Rheum Dis 2009; 68: 784–788. 19147614 10.1136/ard.2008.101501

[68] Sieper J Rudwaleit M Baraliakos X, et al The Assessment of SpondyloArthritis international Society (ASAS) handbook: a guide to assess spondyloarthritis. Ann Rheum Dis 2009; 68(Suppl. 2): ii1–ii44. 10.1136/ard.2008.104018 19433414

[69] Sun L Wu R Xue Q, et al Risk factors of uveitis in ankylosing spondylitis: an observational study. Medicine (Baltimore) 2016; 95: e4233. 10.1097/MD.0000000000004233 PMC4956824 27428230

[70] Maksymowych WP Chou CT Russell AS Matching prevalence of peripheral arthritis and acute anterior uveitis in individuals with ankylosing spondylitis. Ann Rheum Dis 1995; 54: 128–130. 7702400 10.1136/ard.54.2.128 PMC1005535

[71] Singh G Lawrence A Agarwal V, et al Higher prevalence of extra-articular manifestations in ankylosing spondylitis with peripheral arthritis. J Clin Rheumatol 2008; 14: 264–266. 18824928 10.1097/RHU.0b013e31817b8789

[72] Frantz C Portier A Etcheto A, et al Acute anterior uveitis in spondyloarthritis: a monocentric study of 301 patients. Clin Exp Rheumatol 2019; 37: 26–31. 30620268

[73] Gouveia EB Elmann D Morales MS Ankylosing spondylitis and uveitis: overview. Rev Bras Reumatol 2012; 52: 742–756. 23090374

[74] Chen CH Lin KC Chen HA, et al Association of acute anterior uveitis with disease activity, functional ability and physical mobility in patients with ankylosing spondylitis: a cross-sectional study of Chinese patients in Taiwan. Clin Rheumatol 2007; 26: 953–957. 17021671 10.1007/s10067-006-0403-2

[75] Canoui-Poitrine F Lekpa FK Farrenq V, et al Prevalence and factors associated with uveitis in spondylarthritis patients in France: results from an observational survey. Arthritis Care Res (Hoboken) 2012; 64: 919–924. 10.1002/acr.21616 22262475

[76] Pleyer U Pohlmann D Kardes E, et al Emerging drugs for the treatment of noninfectious uveitis. Expert Opin Emerg Drugs 2019; 24: 173–190. 31498689 10.1080/14728214.2019.1663823

[77] Ward MM Deodhar A Gensler LS, et al 2019 update of the American college of rheumatology/spondylitis association of America/spondyloarthritis research and treatment network recommendations for the treatment of ankylosing spondylitis and nonradiographic axial spondyloarthritis. Arthritis Rheumatol 2019; 71: 1599–1613. 31436036 10.1002/art.41042 PMC6764882

[78] van der Heijde D Ramiro S Landewe R, et al 2016 update of the ASAS-EULAR management recommendations for axial spondyloarthritis. Ann Rheum Dis 2017; 76: 978–991. 28087505 10.1136/annrheumdis-2016-210770

[79] Dunne JA Travers JP Double-blind clinical trial of topical steroids in anterior uveitis. Br J Ophthalmol 1979; 63: 762–767. 389282 10.1136/bjo.63.11.762 PMC1043614

[80] Gordon DM Prednisone and prednisolone in ocular disease. Am J Ophthalmol 1956; 41: 593–600. 13302360

[81] Gaudio PA A review of evidence guiding the use of corticosteroids in the treatment of intraocular inflammation. Ocul Immunol Inflamm 2004; 12: 169–192. 15385194 10.1080/092739490500192

[82] Lowder C Belfort R Jr. Lightman S, et al Dexamethasone intravitreal implant for noninfectious intermediate or posterior uveitis. Arch Ophthalmol 2011; 129: 545–553. 21220619 10.1001/archophthalmol.2010.339

[83] Jaffe GJ Martin D Callanan D, et al Fluocinolone acetonide implant (Retisert) for noninfectious posterior uveitis: thirty-four-week results of a multicenter randomized clinical study. Ophthalmology 2006; 113: 1020–1027. 16690128 10.1016/j.ophtha.2006.02.021

[84] Campochiaro PA Brown DM Pearson A, et al Sustained delivery fluocinolone acetonide vitreous inserts provide benefit for at least 3 years in patients with diabetic macular edema. Ophthalmology 2012; 119: 2125–2132. 22727177 10.1016/j.ophtha.2012.04.030

[85] Touhami S Diwo E Seve P, et al Expert opinion on the use of biological therapy in non-infectious uveitis. Expert Opin Biol Ther 2019; 19: 477–490. 30888881 10.1080/14712598.2019.1595578

[86] Kiddee W Trope GE Sheng L, et al Intraocular pressure monitoring post intravitreal steroids: a systematic review. Surv Ophthalmol 2013; 58: 291–310. 23768920 10.1016/j.survophthal.2012.08.003

[87] Haibel H Fendler C Listing J, et al Efficacy of oral prednisolone in active ankylosing spondylitis: results of a double-blind, randomised, placebo-controlled short-term trial. Ann Rheum Dis 2014; 73: 243–246. 23625982 10.1136/annrheumdis-2012-203055

[88] Haroon M Ahmad M Baig MN, et al Inflammatory back pain in psoriatic arthritis is significantly more responsive to corticosteroids compared to back pain in ankylosing spondylitis: a prospective, open-labelled, controlled pilot study. Arthritis Res Ther 2018; 20: 73. 29665824 10.1186/s13075-018-1565-4 PMC5905178

[89] Kroon FP van der Burg LR Ramiro S, et al Nonsteroidal antiinflammatory drugs for axial spondyloarthritis: a Cochrane review. J Rheumatol 2016; 43: 607–617. 26834216 10.3899/jrheum.150721

[90] Fiorelli VMB Bhat P Stephen Foster C Nonsteroidal anti-inflammatory therapy and recurrent acute anterior uveitis. Ocul Immunol Inflamm 2010; 18: 116–120. 20370341 10.3109/09273941003587558

[91] Levinson RD Rosenbaum JT Nonsteroidal anti-inflammatory drugs for prophylaxis of acute anterior uveitis. Ocul Immunol Inflamm 2010; 18: 69–71. 20370330 10.3109/09273941003768224

[92] Lim BX Lim CH Lim DK, et al Prophylactic non-steroidal anti-inflammatory drugs for the prevention of macular oedema after cataract surgery. Cochrane Database Syst Rev 2016; 11: CD006683. 10.1002/14651858.CD006683.pub3 PMC6464900 27801522

[93] Hariprasad SM Akduman L Clever JA, et al Treatment of cystoid macular edema with the new-generation NSAID nepafenac 0.1%. Clin Ophthalmol 2009; 3: 147–154. 19668559 10.2147/opth.s4684 PMC2709014

[94] Petrushkin H Rogers D Pavesio C The use of topical non-steroidal anti-inflammatory drugs for uveitic cystoid macular edema. Ocul Immunol Inflamm 2018; 26: 795–797. 28080174 10.1080/09273948.2016.1269931

[95] Rademacher J Poddubnyy D Emerging drugs for the treatment of axial spondyloarthritis. Expert Opin Emerg Drugs 2018; 23: 83–96. 29475394 10.1080/14728214.2018.1445719

[96] Braun J Zochling J Baraliakos X, et al Efficacy of sulfasalazine in patients with inflammatory back pain due to undifferentiated spondyloarthritis and early ankylosing spondylitis: a multicentre randomised controlled trial. Ann Rheum Dis 2006; 65: 1147–1153. 16606646 10.1136/ard.2006.052878 PMC1798286

[97] Benitez-Del-Castillo JM Garcia-Sanchez J Iradier T, et al Sulfasalazine in the prevention of anterior uveitis associated with ankylosing spondylitis. Eye 2000; 14: 340–343. 11026996 10.1038/eye.2000.84

[98] Munoz-Fernandez S Hidalgo V Fernandez-Melon J, et al Sulfasalazine reduces the number of flares of acute anterior uveitis over a one-year period. J Rheumatol 2003; 30: 1277–1279. 12784403

[99] Munoz-Fernandez S Garcia-Aparicio AM Hidalgo MV, et al Methotrexate: an option for preventing the recurrence of acute anterior uveitis. Eye 2009; 23: 1130–1133. 18688259 10.1038/eye.2008.198

[100] Bachta A Kisiel B Tlustochowicz M, et al High efficacy of methotrexate in patients with recurrent idiopathic acute anterior uveitis: a prospective study. Arch Immunol Ther Exp (Warsz) 2017; 65: 93–97. 27170318 10.1007/s00005-016-0402-1

[101] Zu Hoerste MM Walscheid K Tappeiner C, et al The effect of methotrexate and sulfasalazine on the course of HLA-B27-positive anterior uveitis: results from a retrospective cohort study. Graefes Arch Clin Exp Ophthalmol 2018; 256: 1985–1992. 30069748 10.1007/s00417-018-4082-x

[102] Kempen JH Daniel E Gangaputra S, et al Methods for identifying long-term adverse effects of treatment in patients with eye diseases: the systemic immunosuppressive therapy for eye diseases (SITE) cohort study. Ophthalmic Epidemiol 2008; 15: 47–55. 18300089 10.1080/09286580701585892

[103] Knickelbein JE Kim M Argon E, et al Comparative efficacy of steroid-sparing therapies for non-infectious uveitis. Expert Rev Ophthalmol 2017; 12: 313–319. 30867672 10.1080/17469899.2017.1319762 PMC6411306

[104] Braun J Pham T Sieper J, et al International ASAS consensus statement for the use of anti-tumour necrosis factor agents in patients with ankylosing spondylitis. Ann Rheum Dis 2003; 62: 817–824. 12922952 10.1136/ard.62.9.817 PMC1754665

[105] Davis JC Jr. Van Der Heijde D Braun J, et al Recombinant human tumor necrosis factor receptor (etanercept) for treating ankylosing spondylitis: a randomized, controlled trial. Arthritis Rheum 2003; 48: 3230–3236. 14613288 10.1002/art.11325

[106] van der Heijde D Kivitz A Schiff MH, et al Efficacy and safety of adalimumab in patients with ankylosing spondylitis: results of a multicenter, randomized, double-blind, placebo-controlled trial. Arthritis Rheum 2006; 54: 2136–2146. 16802350 10.1002/art.21913

[107] Inman RD Davis JC Jr. Heijde D, et al Efficacy and safety of golimumab in patients with ankylosing spondylitis: results of a randomized, double-blind, placebo-controlled, phase III trial. Arthritis Rheum 2008; 58: 3402–3412. 18975305 10.1002/art.23969

[108] Braun J Brandt J Listing J, et al Treatment of active ankylosing spondylitis with infliximab: a randomised controlled multicentre trial. Lancet 2002; 359: 1187–1193. 11955536 10.1016/s0140-6736(02)08215-6

[109] Landewe R Braun J Deodhar A, et al Efficacy of certolizumab pegol on signs and symptoms of axial spondyloarthritis including ankylosing spondylitis: 24-week results of a double-blind randomised placebo-controlled phase 3 study. Ann Rheum Dis 2014; 73: 39–47. 24013647 10.1136/annrheumdis-2013-204231 PMC3888598

[110] Sieper J Poddubnyy D New evidence on the management of spondyloarthritis. Nat Rev Rheumatol 2016; 12: 282–295. 27052489 10.1038/nrrheum.2016.42

[111] Jaffe GJ Dick AD Brezin AP, et al Adalimumab in patients with active noninfectious uveitis. N Engl J Med 2016; 375: 932–943. 27602665 10.1056/NEJMoa1509852

[112] Nguyen QD Merrill PT Jaffe GJ, et al Adalimumab for prevention of uveitic flare in patients with inactive non-infectious uveitis controlled by corticosteroids (VISUAL II): a multicentre, double-masked, randomised, placebo-controlled phase 3 trial. Lancet 2016; 388: 1183–1192. 27542302 10.1016/S0140-6736(16)31339-3

[113] Ramanan AV Dick AD Jones AP, et al Adalimumab plus methotrexate for uveitis in juvenile idiopathic arthritis. N Engl J Med 2017; 376: 1637–1646. 28445659 10.1056/NEJMoa1614160

[114] Quartier P Baptiste A Despert V, et al ADJUVITE: a double-blind, randomised, placebo-controlled trial of adalimumab in early onset, chronic, juvenile idiopathic arthritis-associated anterior uveitis. Ann Rheum Dis 2018; 77: 1003–1111. 29275333 10.1136/annrheumdis-2017-212089

[115] Angeles-Han ST Ringold S Beukelman T, et al 2019 American College of Rheumatology/Arthritis Foundation guideline for the screening, monitoring, and treatment of juvenile idiopathic arthritis-associated uveitis. Arthritis Care Res (Hoboken) 2019; 71: 703–716. 10.1002/acr.23871 PMC6777949 31021540

[116] Rudwaleit M Rødevand E Holck P, et al Adalimumab effectively reduces the rate of anterior uveitis flares in patients with active ankylosing spondylitis: results of a prospective open-label study. Ann Rheum Dis 2009; 68: 696–701. 18662932 10.1136/ard.2008.092585 PMC2663712

[117] Van Denderen JC Visman IM Nurmohamed MT, et al Adalimumab significantly reduces the recurrence rate of anterior uveitis in patients with ankylosing spondylitis. J Rheumatol 2014; 41: 1843–1848. 25086071 10.3899/jrheum.131289

[118] Rudwaleit M Rosenbaum JT Landewe R, et al Observed incidence of uveitis following certolizumab pegol treatment in patients with axial spondyloarthritis. Arthritis Care Res (Hoboken) 2016; 68: 838–844. 26815944 10.1002/acr.22848 PMC5089650

[119] Tosi GM Sota J Vitale A, et al Efficacy and safety of certolizumab pegol and golimumab in the treatment of non-infectious uveitis. Clin Exp Rheumatol 2019; 37: 680–683. 30943133

[120] Fabiani C Vitale A Rigante D, et al Efficacy of anti-tumour necrosis factor-alpha monoclonal antibodies in patients with non-infectious anterior uveitis. Clin Exp Rheumatol 2019; 37: 301–305. 30719968

[121] Van der Horst-Bruinsma I van Bentum RE Verbraak FD, et al Reduction of anterior uveitis flares in patients with axial spondyloarthritis following 1 year of treatment with certolizumab pegol: 48-week interim results from a 96-week open-label study. Arthritis Rheumatol 2019; 71(Suppl. 10): Abstract 935.

[122] Braun J Baraliakos X Listing J, et al Decreased incidence of anterior uveitis in patients with ankylosing spondylitis treated with the anti-tumor necrosis factor agents infliximab and etanercept. Arthritis Rheum 2005; 52: 2447–2451. 16052578 10.1002/art.21197

[123] Calvo-Rio V Blanco R Santos-Gomez M, et al Golimumab in refractory uveitis related to spondyloarthritis. Multicenter study of 15 patients. Semin Arthritis Rheum 2016; 46: 95–101. 27060872 10.1016/j.semarthrit.2016.03.002

[124] Van Bentum RE Heslinga SC Nurmohamed MT, et al Reduced occurrence rate of acute anterior uveitis in ankylosing spondylitis treated with golimumab - the GO-EASY study. J Rheumatol 2019; 46: 153–159. 30385705 10.3899/jrheum.180312

[125] Lie E Lindstrom U Zverkova-Sandstrom T, et al Tumour necrosis factor inhibitor treatment and occurrence of anterior uveitis in ankylosing spondylitis: results from the Swedish biologics register. Ann Rheum Dis 2017; 76: 1515–1521. 28254789 10.1136/annrheumdis-2016-210931

[126] Cobo-Ibanez T del Carmen Ordonez M Munoz-Fernandez S, et al Do TNF-blockers reduce or induce uveitis? Rheumatology 2008; 47: 731–732. 18346974 10.1093/rheumatology/ken091

[127] Guignard S Gossec L Salliot C, et al Efficacy of tumour necrosis factor blockers in reducing uveitis flares in patients with spondylarthropathy: a retrospective study. Ann Rheum Dis 2006; 65: 1631–1634. 16901960 10.1136/ard.2006.052092 PMC1798480

[128] Lim LL Fraunfelder FW Rosenbaum JT Do tumor necrosis factor inhibitors cause uveitis? A registry-based study. Arthritis Rheum 2007; 56: 3248–3252. 17907169 10.1002/art.22918

[129] Baeten D Sieper J Braun J, et al Secukinumab, an interleukin-17A inhibitor, in ankylosing spondylitis. N Engl J Med 2015; 373: 2534–2548. 26699169 10.1056/NEJMoa1505066

[130] Deodhar AA Dougados M Baeten DL, et al Effect of secukinumab on patient-reported outcomes in patients with active ankylosing spondylitis: a phase III randomized trial (MEASURE 1). Arthritis Rheumatol 2016; 68: 2901–2910. 27390130 10.1002/art.39805 PMC5132041

[131] Sieper J Deodhar A Marzo-Ortega H, et al Secukinumab efficacy in anti-TNF-naive and anti-TNF-experienced subjects with active ankylosing spondylitis: results from the MEASURE 2 study. Ann Rheum Dis 2017; 76: 571–592. 27582421 10.1136/annrheumdis-2016-210023

[132] Letko E Yeh S Foster CS, et al Efficacy and safety of intravenous secukinumab in noninfectious uveitis requiring steroid-sparing immunosuppressive therapy. Ophthalmology 2015; 122: 939–948. 25638011 10.1016/j.ophtha.2014.12.033

[133] Dick AD Tugal-Tutkun I Foster S, et al Secukinumab in the treatment of noninfectious uveitis: results of three randomized, controlled clinical trials. Ophthalmology 2013; 120: 777–787. 23290985 10.1016/j.ophtha.2012.09.040

[134] Pavelka K Kivitz A Dokoupilova E, et al Efficacy, safety, and tolerability of secukinumab in patients with active ankylosing spondylitis: a randomized, double-blind phase 3 study, MEASURE 3. Arthritis Res Ther 2017; 19: 285. 29273067 10.1186/s13075-017-1490-y PMC5741872

[135] Kim M Won JY Choi SY, et al Anti-TNFα treatment for HLA-B27-positive ankylosing spondylitis-related uveitis. Am J Ophthalmol 2016; 170: 32–40. 27470062 10.1016/j.ajo.2016.07.016

[136] Lian F Zhou J Wei C, et al Anti-TNFα agents and methotrexate in spondyloarthritis related uveitis in a Chinese population. Clin Rheumatol 2015; 34: 1913–1920. 26070537 10.1007/s10067-015-2989-8

[137] Wendling D Joshi A Reilly P, et al Comparing the risk of developing uveitis in patients initiating anti-tumor necrosis factor therapy for ankylosing spondylitis: an analysis of a large US claims database. Curr Med Res Opin 2014; 30: 2515–2521. 25252590 10.1185/03007995.2014.969368

[138] Fouache D Goeb V Massy-Guillemant N, et al Paradoxical adverse events of anti-tumour necrosis factor therapy for spondyloarthropathies: a retrospective study. Rheumatology 2009; 48: 761–764. 19395543 10.1093/rheumatology/kep083

[139] Paley MA Karacal H Rao PK, et al Tofacitinib for refractory uveitis and scleritis. Am J Ophthalmol Case Rep 2019; 13: 53–55. 30582071 10.1016/j.ajoc.2018.12.001 PMC6288302

[140] Askari G Moravejolahkami AR Synbiotic supplementation may relieve anterior uveitis, an ocular manifestation in Behcet’s syndrome. Am J Case Rep 2019; 20: 548–550. 31000688 10.12659/AJCR.912023 PMC6485042

[141] Choi RY Asquith M Rosenbaum JT Fecal transplants in spondyloarthritis and uveitis: ready for a clinical trial? Curr Opin Rheumatol 2018; 30: 303–309. 29538010 10.1097/BOR.0000000000000506

